# Experience of ECMO in Primary Graft Dysfunction after Orthotopic Heart Transplantation

**DOI:** 10.5935/abc.20150082

**Published:** 2015-09

**Authors:** Elson Borges Lima, Claudio Ribeiro da Cunha, Vitor Salvatore Barzilai, Marcelo Botelho Ulhoa, Maria Regina de Barros, Camila Scatolin Moraes, Letycia Chagas Fortaleza, Nubia Wellerson Vieira, Fernando Antibas Atik

**Affiliations:** Instituto de Cardiologia do Distrito Federal, Brasília, DF – Brazil

**Keywords:** Extracorporeal Membrane Oxygenation / methods, Heart Transplantation, Primary Graft Dysfunction / physiopathology, Postoperative Care

## Abstract

**Background:**

Primary graft dysfunction is the main cause of early mortality after heart transplantation. Mechanical circulatory support has been used to treat this syndrome.

**Objective:**

Describe the experience with extracorporeal membrane oxygenation to treat post-transplant primary cardiac graft dysfunction.

**Methods:**

Between January 2007 and December 2013, a total of 71 orthotopic heart transplantations were performed in patients with advanced heart failure. Eleven (15.5%) of these patients who presented primary graft dysfunction constituted the population of this study. Primary graft dysfunction manifested in our population as failure to wean from cardiopulmonary bypass in six (54.5%) patients, severe hemodynamic instability in the immediate postoperative period with severe cardiac dysfunction in three (27.3%), and cardiac arrest (18.2%). The average ischemia time was 151 ± 82 minutes. Once the diagnosis of primary graft dysfunction was established, we installed a mechanical circulatory support to stabilize the severe hemodynamic condition of the patients and followed their progression longitudinally.

**Results:**

The average duration of extracorporeal membrane oxygenation support was 76 ± 47.4 hours (range 32 to 144 hours). Weaning with cardiac recovery was successful in nine (81.8%) patients. However, two patients who presented cardiac recovery did not survive to hospital discharge.

**Conclusion:**

Mechanical circulatory support with central extracorporeal membrane oxygenation promoted cardiac recovery within a few days in most patients.

## Introduction

Primary graft dysfunction (PGD) is a syndrome of cardiac dysfunction that occurs in the immediate postoperative period after cardiac transplantation and is the main isolated cause of death within the first 30 days after transplantation^1^. The etiology of PGD includes factors inherent to the recipient, donor, and perioperative care. The control of all the factors that can lead to this catastrophic complication is challenging.

The prevalence of PGD ranges from 2.3 to 28% when experiences of isolated centers are analyzed^2-4^. These differences in prevalence are due in part to the lack of systematization of the diagnostic criteria, which are based on individual definitions adopted by each transplanting center. The most controversial definition factors in PGD include time of onset, echocardiographic findings, hemodynamic measures, requirement of mechanical circulatory support, and exclusion factors such as rejection. Based on these challenges, the International Society for Heart & Lung Transplantation recently organized a consensus in order to standardize the definition, diagnosis and management of PGD after cardiac transplantation^5^.

The knowledge of probable risk factors in PGD led to the development of a risk score^6^ which has been validated in other populations for an adequate assessment of the syndrome. Preventive recommendations regarding donor management and for the perioperative period have been recently published in an expert consensus^5^ in order to minimize the occurrence of PGD.

The initial treatment of PGD is clinical. Mechanical circulatory support, in turn, is recommended early in the most severe cases, and usually involves circulatory assistance with extracorporeal membrane oxygenation (ECMO) or with a ventricular assist device. Experiences from several centers^7,8^ suggest improvement in early and late survival with this strategy. In our environment, the use of ECMO for resuscitation of severe hemodynamic disorders has been limited^9^, and its application in the postoperative period of cardiac transplantation lacks description in the national literature.

The aim of this study was to describe the experience with ECMO for the treatment of post-transplant cardiac PGD.

## Methods

Between January 2007 and December 2013, a total of 71 heart transplantations were performed in patients with advanced heart failure. Eleven (15.5%) of these patients presented PGD and comprised the population of our study. Their average age was 33.8 ± 20.7 years (range 16 to 63 years) and seven (63.6%) were male.

The definition of post-transplantation PGD was based on the recent consensus of the International Society for Heart & Lung Transplantation^5^, defined as any graft dysfunction that occurs up to 24 hours after the transplantation. PGD was also classified into left ventricular (LV) PGD, which included biventricular dysfunction with three degrees of severity, and in right ventricular (RV) PGD. The exact definitions are shown in chart 1. We excluded from the definition secondary causes of graft dysfunction, such as hyperacute rejection, pulmonary hypertension, or surgical complications.

Our patients presented severe hemodynamic instability, which occurred in the initial 24 hours after the surgery and was secondary to cardiac dysfunction documented by echocardiography. The hemodynamic instability was necessarily unresponsive to volume replacement, rhythm control, and use of two inotropes, and was characterized by low cardiac output (cardiac index < 2 L/min/m^2^), with elevation in filling pressures (pulmonary capillary pressure > 20 mmHg or central venous pressure > 15 mmHg) in the absence of pulmonary hypertension with isolated secondary RV dysfunction. Thereby, all patients fit into the classification of severe PGD-LV (Chart 1).

**Chart 1:** Classification of primary graft dysfunction after heart transplantation^5^

PGD-LV	Mild - meets one of the following criteria:	Echocardiography: LVEF < 40% OR Hemodynamics: CVP > 15 mmHg, PCWP > 20 mmHg, CI < 2 L/min/m^2^ lasting for 1 hour and requiring low-dose inotropes
	Moderate - meets one criterion from 1 and another criterion from 2:	1.Echocardiography: LVEF < 40% OR Hemodynamics: CVP > 15 mmHg, PCWP > 20 mmHg, CI < 2 L/min/m^2^, hypotension with MAP < 70 mmHg 2.Inotrope score > 10 or intra-aortic balloon pump
PGD-RV	Severe	Dependence on mechanical circulatory support, excluding intra-aortic balloon pump
	Requires 1 + 2, or 3 alone	1.CVP > 15 mmHg, PCWP < 15 mmHg, CI < 2 L/min/m^2^ 2.TPG < 15 mmHg AND/OR SBP < 50 mmHg 3.Requirement of right circulatory assistance

PGD-LV: left ventricular primary graft dysfunction; LVEF: left ventricular ejection fraction; CVP: central venous pressure; PCWP: pulmonary capillary wedge pressure; CI: cardiac index; MAP: mean arterial pressure; PGD-RV: right ventricular primary graft dysfunction; TPG: transpulmonary pressure gradient; SBP: systolic blood pressure.

The detailed characteristics of the recipients and donors are shown in Table 1. All patients had advanced heart failure, which was categorized as functional class III in six patients and class IV in five patients. Two (18.2%) patients were in a priority state before the transplantation and were receiving intravenous inotropes. As for the etiology of the cardiomyopathy, five (45.4%) were due to Chagas disease, three to idiopathic dilated cardiomyopathy, one was secondary to valvular cardiomyopathy, one to restrictive cardiomyopathy, and another one was associated with peripartum cardiomyopathy. Eight (72.7%) patients received prior to the transplantation an implantable cardiac defibrillator for secondary prevention of sudden death, and one (9.1%) had undergone previous heart surgery.

**Table 1:** Preoperative characteristics of the recipients and donors of patients who progressed with primary graft dysfunction after cardiac transplantation

**Recipients data**	
Age (years)	33.8 ± 20.7
Male gender, n (%)	7 (63.6)
Weight (kg)	51.5 ± 17.7
Height (cm)	157.8 ± 27.4
Race, n (%)	
White	7 (63.6)
Hybrid (Black/White)	3 (27.3)
Black	1 (9.1)
Blood type, n (%)	
O	6 (54.5)
A	3 (27.3)
AB	2 (18.2)
Functional class (NYHA)	
III	6 (54.4)
IV	5 (45.5)
Previous cardiac surgery	1 (9.1)
Previous stroke	1 (9.1)
Systemic arterial hypertension	1 (9.1)
Diabetes mellitus	2 (18.2)
Implantable cardioverter	8 (72.7)
Echocardiographic data	
Ejection fraction (%)	28.5 ± 14.5
Diastolic LV diameter (mm)	61.3 ± 11.1
Systolic LV diameter (mm)	53.8 ± 13.1
Left atrial volume (mL)	97.9 ± 53.3
Hemodynamic data	
Pulmonary systolic pressure (mmHg)	44.6 ± 11.9
Pulmonary vascular resistance (Wood)	2.2 ± 1.45
**Donors data**	
Age (years)	26.6 ± 12.4
Male gender	7 (63.6)
Weight (kg)	60.3 ± 17
Height (cm)	165.4 ± 19
Race	
White	8 (72.7)
Hybrid (Black/White)	3 (27.3)
Blood type	
O	8 (72.7)
A	2 (18.2)
B	1 (9.1)

NYHA: New York Heart Association; LV: left ventricle.

On preoperative echocardiography, the average ejection fraction was 28.5 ± 14.5% (range 14 to 32%) and the diastolic LV diameter was 61.3 ± 11.1 mm (range 37 to 74 mm). Right cardiac catheterization revealed an average pulmonary vascular resistance of 2.2 ± 1.5 Wood units (range 0.5 to 4.6 Wood units). None of the patients had preformed antibodies with titles above 10%.

Donors, which were predominantly male (n = 7; 63.6%), had an average age of 26.6 ± 12.3 years (range 15 to 48 years). The causes of death among donors included head trauma in seven cases (63.6%), hemorrhagic stroke in three (27.3%), and cerebral tumor in one. Seven (63.6%) were receiving continuous infusion of norepinephrine > 0.1 mcg/kg/min at the time of the retrieval.

Retrievals took place in the same hospital of the implantation in six (54.5%) cases and at a distant center in the five remaining cases. These last were retrieved in the states of Goiás, Minas Gerais, São Paulo, Paraná, and Rio Grande do Sul.

The donor's heart was protected with the cardioplegic solution of St. Thomas. The hearts were transported in sterile plastic bags filled with iced saline solution and packed in thermal refrigerators with ice. Intra- and postoperative management protocols were those standardized in our institution and were uniform for all patients. The transplantations were performed after median sternotomy, systemic heparinization, and cardiopulmonary bypass during mild hypothermia with modified hemofiltration. The implantation followed the bicaval technique in all patients. Myocardial protection was achieved with anterograde infusion of St. Thomas solution at 4ºC every 15 minutes during implantation. The average ischemia time was 151 ± 82 minutes (range 73 to 270 minutes), with 82.8 ± 14.2 minutes in the local retrievals and 233 ± 35.4 minutes in distant retrievals (p < 0.0001). The cold ischemia "limit" of 4 hours was exceeded in two (18.2%) patients.

Once the diagnosis of PGD was established, mechanical circulatory support was initiated to stabilize the hemodynamic and/or severe respiratory condition.

The circuit materials, modes of cannulation, and the ECMO protocol followed those described in the specific literature^10,11^. In summary, the ECMO circuit included a polymethylpentene hollow-fiber membrane oxygenator, centrifugal pump, and tubing coated with antiplatelet agents. All patients were treated with the same equipment during the study. The cannulation was preferably central, in the ascending aorta and right atrium in nine (81.8%) patients. Cannulation of the left atrium was recommended in the absence of adequate decompression of the left chambers assessed by echocardiography and direct measurement of pressure in the left atrium. This was a requirement in all patients undergoing central ECMO. The two remaining patients underwent ECMO implantation via femoral vessels.

Heparin was administered continuously prior to cannulation when the activated coagulation time reached 250 seconds, in order to maintain it between 150 and 200 seconds. All patients were maintained sedated, under mechanical ventilation with an orotracheal tube, and with the sternum opened. A silicone membrane was stitched to the edges of the wound, or the skin was simply closed. Concomitant intra-aortic balloon pump was used routinely for hemodynamic support during weaning and was recommended as soon as early signs of function recovery were detected on echocardiography. When the intra-aortic balloon pump had already been implanted prior to the ECMO, it was maintained for support. Cardiopulmonary support was maintained with the intentions of recovery and weaning, according to daily clinical and echocardiographic criteria. The support was discontinued in patients deemed unable to recover and with limited survival according to multidisciplinary judgement.

The manifestations of PGD in our population included failure to wean from cardiopulmonary bypass in six (54.5%) patients, severe hemodynamic instability in the immediate postoperative period with severe cardiac dysfunction in three (27.3%) patients, and cardiac arrest in two (18.2%) patients. In particular, patients with postcardiotomy syndrome showed severe biventricular dysfunction on echocardiography, received maximum doses of at least two positive inotropes, and were treated with intra-aortic balloon pump. These patients had no pulse pressure or ventricular ejection curve on echocardiography. Patients with postoperative hemodynamic instability, in turn, presented macro-hemodynamic parameters compatible with cardiogenic shock (cardiac index < 2 L/min/m^2^, pulmonary capillary pressure > 20 mmHg, central venous pressure > 15 mmHg), despite maximum doses of at least two inotropes, echocardiographic documentation of systolic ventricular dysfunction (LV ejection fraction < 40%), and hypotension requiring vasopressors. The two patients in whom ECMO was installed for cardiopulmonary resuscitation progressed in the hours following cardiac arrest with cardiogenic shock, for which management was initially conservative, but proved to be ineffective.

The pre-, intra-, and postoperative characteristics of the patients were collected prospectively and stored in an electronic database. The clinical progression of the patients was followed longitudinally. The study was approved by the Ethics and Research Committee (CAAE 27039514.5.0000.0026), in accordance with the Declaration of Helsinki.

### Statistical analysis

Categorical variables were expressed as frequencies and percentages, and continuous variables as means and standard deviations. The comparison of categorical variables was carried out with the chi-square test and the comparison of continuous variables with Student's *t* test. The actuarial survival rate was determined by the Kaplan-Meier method. The level of statistical significance was set at 5%, and the statistical software used was JMP for SAS, version 9.

## Results

The average duration of the ECMO assistance was 76 ± 47.4 hours (range 32 to 144 hours). Weaning with cardiac recovery was successful in nine (81.8%) patients. However, two patients who had cardiac recovery did not survive to hospital discharge. One patient had complications related to hemorrhagic stroke and another related to multiple organ failure. Hospital mortality was 36.4%.

The main morbidities, with their respective frequencies, are listed in Table 2. The main problems in postoperative care in these patients were acute renal failure requiring hemodialysis, stroke, surgical revision of hemostasis, and pneumonia. Of the four patients who developed a stroke, only one was discharged from the hospital with a motor deficit in a lower limb which recovered 6 months later with specialized physical therapy. Of the seven patients who required hemodialysis, four were discharged from the hospital with normal renal function, and the other three died.

**Table 2:** Postoperative mortality and morbidity of patients who underwent heart transplantation, progressed with primary graft dysfunction and received extracorporeal membrane oxygenation

	**n (%)**
Hospital mortality	4 (36.4)
Stroke	4 (36.4)
Surgical intervention for hemostasis	4 (36.4)
Sepsis	3 (27.3)
Acute renal failure	7 (63.6)
Prolonged mechanical ventilation	-
Mediastinitis	-
Permanent pacemaker	1 (9.1)
Length of intensive care (days)*	8.5 (5.25 - 10.75)
Length of hospital stay (days)*	22.5 (5.75 - 45.25)

Median (confidence interval 95%)

The immunosuppression differed from that in other patients who did not develop graft dysfunction. Patients on ECMO received routinely induction therapy with thymoglobulin associated with corticosteroids. Other patients were treated with a triple scheme comprised of a calcineurin inhibitor (cyclosporine or tacrolimus), mycophenolate mofetil, and corticosteroids. Endomyocardial biopsy, performed routinely on the seventh day after surgery, revealed that only one (9.1%) patient had progressed with cellular rejection greater than 2R, which excluded the possibility of hyperacute rejection as a secondary cause of cardiac dysfunction. None of the patients underwent plasmapheresis, and analysis of humoral rejection by immunofluorescence, including analysis of complement fragments, was negative in all of them.

The 30-day mortality in our unit was 9.8%, and was higher in patients who progressed with PGD (36.4%) when compared with those who did not present this complication (5%; p = 0.02). None of the patients who survived to hospital discharge died during late follow-up. Actuarial survival at 3 months, 2 years and 3 years was 72.7%, 60.6%, and 60.6%, respectively.

Although the denominator for this analysis is small, weaning from ECMO was successful in all patients in whom ECMO was installed due to postcardiotomy shock in the operating room and postoperative hemodynamic instability, in contrast to those in whom it was installed after cardiac arrest (p = 0.004). Similarly, hospital mortality was greater after cardiac arrest (100%) and postoperative hemodynamic instability (66.7%) than after cardiotomy (0%; p = 0.01).

## Discussion

The present study sought to examine the occurrence of PGD, a serious complication after cardiac transplantation. Although there may be questions regarding its exact definition^12^, we applied recent diagnostic criteria^5^ and found a prevalence of PGD of 15.5% in our population. This rate is in line with rates of other studies in the literature, which range from 2 to 26%^1,5,8^. Despite attempts to control the most frequent risk factors associated with the occurrence of PGD after cardiac transplantation^6^, the occurrence of this complication remains high. However, preventive strategies remain and have focused on better donor choice and maintenance, heart preservation methods in long-distance retrievals with prolonged ischemia time, and better myocardial protection during implantation, among others.

PGD is the main cause of early mortality after transplantation. Hemodynamic deterioration caused by cardiogenic shock due to pump failure unresponsive to inotropes has a catastrophic progression if not corrected in time. For this reason, transplanted patients in our unit are routinely monitored during and after surgery with a Swan-Ganz continuous cardiac output catheter, which provides real-time hemodynamic parameters required for best therapeutic decision-making in combination with other tissue perfusion variables. Intraoperative transesophageal echocardiography is routinely used upon discontinuation of cardiopulmonary bypass to provide information regarding dimensions and biventricular function, estimate blood volume, identify eventual residual surgical defects, and check the function of the heart valves. If the diagnosis of postoperative shock is challenging, bedside echocardiography is very informative and should be performed whenever necessary, mainly when cardiac dysfunction with hemodynamic parameters indicative of cardiogenic shock is suspected.

Cases of severe PGD, such as those presented in Table 1, without response to inotropes and heart rhythm control and in the absence of cardiac tamponade should be treated promptly with mechanical circulatory support. ECMO should be installed early^13^, before the occurrence of multiorgan dysfunction or prior to cardiac arrest, as highlighted in the literature^14^. As described in our experience, the earlier the implantation (operation room), the best are the outcomes for weaning and survival. Patients in whom ECMO was initiated due to cardiac arrest had a poor outcome, and the appropriate timing was certainly neglected.

The aim of circulatory assistance in PGD is always cardiac recovery. Thus, the characteristics of the ideal device must comply with the following requirements: ability to be quickly installed, allow rapid re-establishment of cardiac output in order to maintain adequate tissue perfusion and reverse multiorgan dysfunction, reduce ventricular filling pressures, promote myocardial protection with increased coronary flow and reduced consumption of oxygen, and have a low complication rate.

Heart decompression is important for a successful recovery since intracavitary hypertension curtails subendocardial coronary perfusion. Most of our patients had no electrical activity and lacked sufficient contractile activity for adequate LV decompression. After documentation of high pressures in the left atrium with distension of the LV, we inserted another drainage cannula in the left atrium which controlled or even interrupted the flow depending on the recovery of the LV function. The use of intra-aortic balloon pump has documented benefit in reducing the systemic vascular resistance, which assists in left ventricular recovery and is routine in ECMO weaning.

In our experience, the use of ECMO met the desired goals, promoting cardiac recovery in most cases, with acceptable complication rates, considering the severity of the clinical condition of the patients. We achieved success in removing the ECMO, with cardiac recovery in 81.8% of the patients after an average of 76 hours. These results are in line with those of other international centers^8,14-16^. The main postoperative problems that we found were acute renal failure, stroke, and requirement for surgical revision of hemostasis. The first is a frequent complication^14^ and is secondary to multiple insulting factors (shock, nephrotoxic drugs, and systemic venous congestion) and pretransplant cardiorenal syndrome. Renal function recovered in all patients after a few sessions of hemodialysis, as described by Listijono et al.^15^. The last two are complications related to the requirement of anticoagulation during ECMO, which is difficult to control. Complicating factors are recent heart surgery, presence of shock with concomitant hepatic dysfunction, and, eventually, disseminated intravascular coagulation. Excessive use of blood products complicates immunological sensitization, systemic congestion (including liver congestion, which increases bleeding), and pulmonary hypertension. The use of heparin-coated circuits, antifibrinolytics, adoption of a careful surgical technique, maintenance of hemodynamic stability with systemic venous decompression and synthetic derivatives of coagulation factors are important to minimize these complications. Such measures have been routinely used in our unit. Patients who developed stroke showed increased hospital mortality; those who survived showed full recovery of motor activity without limitation in quality of life.

In our experience, the causes of PGD showed no association with severe rejection, as documented by routine endomyocardial biopsy in the first week, with only one patient presenting cellular rejection greater than 2R. Regardless of showing complete recovery, which occurred in our patients who weaned from ECMO, these patients had severe conditions and higher mortality when multiple organs were involved. This was demonstrated in our experience and is in line with experiences of others with patients treated after postcardiotomy shock^16^. Hence, there is need for strengthened intensive care in this population, systematically focused on the management of organs and systems and on the prevention of sepsis.

Although the experience with ECMO in PGD is well established in the literature, some groups^17,18^ describe the use of short-term ventricular assist devices as a therapeutic option. The advantages of ECMO include biventricular support in all cases, pulmonary support, less thromboembolic complications, and fast installation. Last but not least, especially in our environment, is the fact that the cost of ECMO is much lower than the cost of other ventricular assistance devices.

This descriptive work has some limitations, including the low number of patients in the study group and absence of a control group. We consider that the inclusion of a control group would stumble into important ethical dilemmas, since we would be unable to use a comparison group in which ECMO is not used, due to the high mortality associated with clinical treatment alone. Currently, we do not have other ventricular assist devices to compare with ECMO. We did not list or analyze the risk factors of PGD after heart transplantation, which will be the aim of a future study when we obtain a larger number of patients for more robust statistical analysis.

## Conclusion

Use of mechanical circulatory support with central extracorporeal membrane oxygenation promoted cardiac recovery within a few days in most patients who presented primary graft dysfunction after cardiac transplantation.
